# Injectable systems of chitosan in situ forming composite gel incorporating linezolid-loaded biodegradable nanoparticles for long-term treatment of bone infections

**DOI:** 10.1007/s13346-023-01384-x

**Published:** 2023-08-04

**Authors:** Reem Khaled Wassif, Seham A. Elkheshen, Rehab Nabil Shamma, Mohammed S. Amer, Rehab Elhelw, Maha El-kayal

**Affiliations:** 1 Department of Pharmaceutics and Pharmaceutical Technology, Faculty of Pharmacy, Future University in Egypt, Cairo, Egypt; 2 Department of Pharmaceutics and Industrial Pharmacy, Faculty of Pharmacy, Cairo University, Kasr Elini Street, Cairo, 11562 Egypt; 3 Department of Surgery, Anaesthesiology and Radiology, Faculty of Veterinary Medicine, Cairo University, Cairo, Egypt; 4 Department of Microbiology and Immunology, Faculty of Veterinary Medicine, Cairo University, Cairo, Egypt

**Keywords:** Linezolid, Chitosan in situ forming gel, Biodegradable nanoparticles, Bone infections, Osteomyelitis

## Abstract

The objective of the current study was to create an efficient, minimally invasive combined system comprising in situ forming hydrogel loaded with both spray-dried polymeric nanoparticles encapsulating linezolid and nanohydroxyapatite for local injection to bones or their close vicinity. The developed system was designed for a dual function namely releasing the drug in a sustained manner for long-term treatment of bone infections and supporting bone proliferation and new tissues generation. To achieve these objectives, two release sustainment systems for linezolid were optimized namely a composite in situ forming chitosan hydrogel and spray-dried PLGA/PLA solid nanoparticles. The composite, in situ forming hydrogel of chitosan was prepared using two different gelling agents namely glycerophosphate (GP) and sodium bicarbonate (NaHCO_3_) at 3 different concentrations each. The spray-dried linezolid-loaded PLGA/PLA nanoparticles were developed using a water-soluble carrier (PVP K30) and a lipid soluble one (cetyl alcohol) along with 3 types of dl-lactide and/or dl-lactide-co-glycolide copolymer using nano-spray-drying technique. Finally, the optimized spray-dried linezolid nanoparticles were incorporated into the optimized composite hydrogel containing nanohydroxy apatite (nHA). The combined hydrogel/nanoparticle systems displayed reasonable injectability with excellent gelation time at 37 °C. The optimum formulae sustained the release of linezolid for 7–10 days, which reveals its ability to reduce the frequency of injection during the course of treatment of bones infections and increase the patients’ compliance. They succeeded to alleviate the bone infections and the associated clinical, biochemical, radiological, and histopathological changes within 2–4 weeks of injection. As to the state of art in this study and to the best of our knowledge, no such complete and systematic study on this type of combined in situ forming hydrogel loaded with spray-dried nanoparticles of linezolid is available yet in literatures.

**Graphical Abstract:**

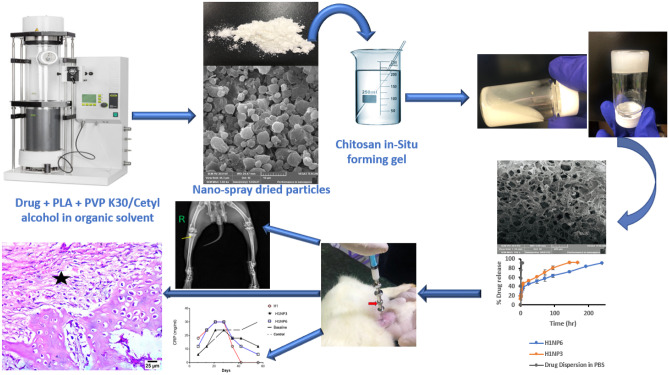

**Supplementary Information:**

The online version contains supplementary material available at 10.1007/s13346-023-01384-x.

## Introduction

Due to the elevated morbidity rates in patients suffering from osteomyelitis and the difficulty of the treatment regimen required to completely eradicate bone infections, significant efforts are being invested in researches to improve the therapeutic outcomes of the local treatment of acute and chronic osteomyelitis [1, 2]. Local delivery of antibiotics has attracted great attention recently, where local application of specialized drug carriers system exhibits several benefits including (1) improving the feasibility of direct penetration of the antimicrobial agent into bone tissues, (2) offering localized and sustained release antimicrobial agents to the infected area, and (3) avoiding the possible side effects and drug-related toxicity accompanying the systemic treatment [3].

Although glycopeptide antibiotics, such as vancomycin, have been traditionally the antibiotics of choice for the treatment of multi-resistant Gram-positive bacteria such as methicillin-resistant *Staphylococcus aureus* (MRSA), they suffer from several drawbacks including the difficulty in achieving appropriate serum levels and the poor penetration into the infected tissues and being subjected to the development of glycopeptide-resistant strains. Linezolid, an antibacterial agent from the oxazolidinones family, is active against Gram-positive bacteria including MRSA and enterococci, which are the major cause of osteomyelitis. Additionally, they are also active against strains resistant to vancomycin [4, 5].

Long-term systemic treatment with linezolid has been linked to toxic optic neuropathy as well as bone marrow suppression, which can lead to anemia, leucopenia, and thrombocytopenia [6, 7]. Therefore, sustained release systems for local delivery of linezolid to the infected bone or their close vicinity can be beneficial for the treatment of acute or chronic osteomyelitis caused by Gram-positive bacteria. Additionally, if made injectable and biodegradable, it can overcome the need for surgeries for both applying and removing the polymeric delivery system [1].

Scaffolds for the local application and antibiotic delivery to bone tissues have been developed using different materials including metals, bio-ceramics, polymers, and polymers composite with bioactive materials and polymers combined with particulate systems [8]. Composite scaffolds made from polymers and ceramics or bioactive glass have been identified as potential biomaterials, which have the ability to fill, replace, and regenerate the injured bone. They show remarkable bioactivity, biocompatibility, injectability, and biodegradability [9]. During the process of bone regeneration after injury or inflammation, a gel scaffold with characteristics similar to the physical and chemical structure of bone matrix is very desirable in order to maintain cell proliferation and differentiation. Integration of natural polysaccharides such as chitosan and inorganic components such as hydroxyapatite nanoparticles (nHA) is the most efficient technique to create these types of gel composite scaffolds [10, 11]. The incorporation of nHA in bone scaffolds with or without natural polymers is very well known to be beneficial for the process of bone regeneration due to the similarity of nHA to the natural extracellular matrix (ECM) of bones, together with its ability to increase the mechanical strength of the formed gel [12–14]. Thermosensitive systems have been proven to be crucial since they demonstrate sol–gel transition without the use of organic solvents or harmful cross-linking agents [15]. In situ forming hydrogel can form under the influence of pH and temperature changes [16]. Studies have reported the use of sodium bicarbonate (NaHCO_3_) as a neutralizing agent to chitosan solution to form a three-dimensional network upon increasing the temperature of the solution to the body temperature while being at pH above the pKa of chitosan [17, 18]. Furthermore, the reaction of chitosan and polyol-phosphate salts such as glycerol, sorbitol, fructose, and glucose phosphate salts can form hydrogel through hydrogen bonding, electrostatic, and hydrophobic interactions if heated at body temperature [16, 19].

Combining hydrogel scaffold and/or composite scaffold with particulate system can offer the advantages of both systems. Scaffold becomes osteoconductive through occupying the dead space in the bone structure and enhancing the process of osteogenesis specially if containing a bioactive material, while the particulate system can deliver the antibiotic in a sustained release kinetics without being dependent on the rate of degradation of the scaffold [20, 21]. In earlier studies, the use of PLGA polymer proved to be promising approach to develop sustained release drug delivery systems for highly water-soluble drugs [22–24]. Being biocompatible and biodegradable makes this category of polymers the perfect choice for injectable polymeric systems to avoid tissues interactions or the need to remove the delivery system after drug depletion.

From a clinical point of view, there are several commercially available scaffolding materials either incorporating antimicrobial agents (gentamicin, tobramycin, clindamycin, and vancomycin) or ready to be loaded with them in-suit before application. These carriers are usually applied as a cement on the bone surface after debridement of the deteriorated bone and some of them may need another surgery to remove the cement. They include polymethylmethacrylate, hydroxyapatite, calcium sulfate, calcium phosphate, collagen, and their composites. These commercial products have been approved by different regulatory bodies all over the world [1]. Furthermore, ongoing clinical investigations in the field of orthopedics on the local application of more vehicles loaded with antibiotics along with bone filling materials for the treatment of long bone infections are also available. They proved their efficacy in early suppression of infection, significant reduction in recurrent infection rates, decreased risk of pathological fracture due to internal reinforcement, prompt recovery of extremity function, and creation of a favorable environment for bone structure restoration [25–27]. All clinically applied or investigated cements require direct application on the bone surface after surgery.

In the early stages of bone infections, surgery may not be needed so the systemic treatment is the only solution with all its drawbacks specially if needed for long period of time. Some investigations in animals are available for the delivery of therapeutic agents using injectable in suite forming scaffolds to control inflammation and enhance new bone regeneration without performing surgery and most of them had beneficial effects [17, 28–30].

The novelty of the current study lies in developing a delivery system for linezolid that overcome its side effect if given systemically for long-term treatment of bone infection, utilizing the most recent ideas for developing locally injectable sustained release in situ forming scaffold using combined particulate/hydrogel system made of biodegradable nanoparticles incorporating the drug which are suspended in dispersion that allow the formation of hydrophilic composite gel for both treatment of bone infection and assistance in the regeneration of new tissues through the presence of nHA. The developed system also allows multiple local administration to the infected bones or their close vicinity without having to cut open the patient. The optimized systems will be tested in animal model.

## Materials and methods

### Materials

Linezolid was a kind gift from Global Napi Pharmaceuticals, Egypt. Chitosan medium molecular weight, nanohydroxyapatite (nHA), glycerophosphate disodium salt hydrate (GP), polyvinylpyrrolidone (PVP) K30, and dialysis cellulose membrane (molecular weight cutoff 14,000 g/mol) were purchased from Sigma-Aldrich, USA. Poly-dl-lactide/glycolide 75:25 copolymer, end-capped with ester terminal and having inherent viscosity of 0.22 dL/g (PLGA, PURASORB PDLG 7502); poly-dl-lactide/glycolide 85:15 copolymer, end-capped with ester terminal and having inherent viscosity of 0.22 dL/g (PLGA, PURASORB PDLG 8502); and poly-dl-lactide polymer, end-capped with ester terminal and having inherent viscosity of 0.20 dL/g (PLA, PURASORB PDL 02) were kindly donated by PURAC Biomaterials, Gorinchem, Netherlands. Cetyl alcohol was supplied by BioTech for laboratory chemicals, Egypt. Acetonitrile and acetone were purchased from Thermo Fisher Scientific, UK. Sodium bicarbonate, acetic acid, disodium hydrogen phosphate, sodium chloride, and potassium dihydrogen phosphate were obtained from ADWIC, El-Nasr Pharmaceutical Chemicals Co., Egypt. Betolvex^®^ oily injection, batch number MFE0841, was purchased from MinaPharm Pharmaceuticals, Egypt. Xyla-Ject was purchased from Adwia Pharmaceuticals, Egypt, and ketamine from Alfasan International, Holland.

### Methods

#### Preparation of in situ forming composite hydrogel systems loaded with linezolid and nanohydroxyapatite

Preparation of the composite hydrogels was conducted as described previously [17]. Briefly, 2% (w/v) solution of chitosan was prepared by dissolving accurately weighed amount of chitosan in 1% (v/v) acetic acid via stirring for 2 h at 250 rpm using magnetic stirrer (VELP-AREC.T, Italy). One percent w/v of each of linezolid and nanohydroxyapatite (nHA) was added to the chitosan solution on the magnetic stirrer and stirred for 15 min to obtain homogeneous dispersion. Two gelling agents for chitosan, each in three different concentrations namely NaHCO_3_ (1%, 2%, and 4%, in volume ratio of 1:1 of NaHCO_3_:chitosan solutions) and glycerophosphate (GP) (25%, 50%, and 75%, in volume ratio of 1:9 of GP:chitosan solutions) were used as previously reported [17, 19]. Solutions of NaHCO_3_ or GP were added drop wise to chitosan solution on a magnetic stirrer until a homogeneous dispersion was obtained. The compositions of the successfully performing formulations are presented in Table 1.

**Table 1:** The composition and characterization of hydrogel formulations containing free drug and nHA (Data are mean ± SD, n = 3)

Hydrogel formulae-code	Composition of the hydrogel systems						Characterization of the hydrogel systems						
	Linezolid (mg/mL)	nHA (mg/mL)	Chitosan (%w/v)	Concentration of gelling agent		Chitosan: gelling agent solutions- ratio (v/v)	Gelation time (min.)	Viscosity (cP)				t_80%_ (hr)	Desirability
				NaHCO_3_ (%w/v)	GP (%w/v)			Liquid at 25 °C		Gel At 37 °C			
								10 rpm	250 rpm	10 rpm	250 rpm		
H1	10	10	2	2	-	1:1	5.0 ± 0.00	204.4 ± 2.47	49.39 ± 6.8	3773.76 ± 4.81	282.45 ± 4.31	16.87 ± 1.11	0.724
H2	10	10	2	4	-	1:1	3.5 ± 0.00	207.77 ± 5.26	50.34 ± 8.14	5320.09 ± 2.69	293.55 ± 2.05	18.71 ± 0.38	0.000
H3	10	10	2	-	50	9:1	6.5 ± 0.00	242.86 ± 3.73	60.32 ± 5.39	895.63 ± 5.04	114.2 ± 8.93	7.963 ± 0.28	0.000
H4	10	10	2	-	75	9:1	5.0 ± 0.70	244.595 ± 1.84	62.4 ± 2.46	933.15 ± 2.61	131.25 ± 8.13	8.877 ± 0.25	0.000

#### Evaluation of the prepared in situ forming hydrogel systems

##### In vitro gelation time

The gelation time of different hydrogel formulations using NaHCO_3_ or GP as gelling agent was estimated using the test tube inversion method as previously described [31]. Two milliliters of the prepared mixtures were incubated at 37 °C in a thermostatically controlled water bath. The test tubes were turned over every 30 s to check the flowability of the solutions. The gelation time was considered the time required to create a gel that does not flow within 5 s of the test tube inversion.

##### Rheological properties

The rheological behavior of different hydrogel formulations was evaluated using Brookfield viscometer (model DV3THBCJO, USA). Briefly, 1 mL of each of the tested formulations was placed on the plate of the viscometer and sheared at speed settings of 10 rpm up to 250 rpm (equivalent to shear rate output of 75 to 1875s^−1^). The experiment was conducted at 25 °C ± 0.2 °C for the fresh dispersion and at 37 °C ± 0.2 °C for the gelatinized dispersion. The flow behavior of the tested formulations was estimated by plotting the logarithmic values of the shear stress (F) against the shear rate (G) applying Farrow’s equation:

1 $$\mathrm{Log\;G=N\;Log\;F-Log\;\eta}$$

where G is the shear rate (s^−1^), F is the shear stress (dyne/cm^2^), η is the viscosity (cP), and N is Farrow’s constant. Based on the previous equation, Newtonian flow is revealed when *N* = 1, while dilatant flow is revealed at *N* < 1 (i.e., shear thickening behavior), whereas pseudoplastic or plastic flow is revealed when *N* > 1 (i.e., shear thinning) as previously mentioned [32, 33].

##### In vitro release of linezolid from the composite hydrogel systems

The in vitro release testing of linezolid from the prepared hydrogel formulations was carried out using the dialysis cellulose membrane diffusion technique as reported previously [34]. Dialysis bags (7-cm length each) were pre-soaked for 12 h in the release medium (phosphate buffer saline, pH = 7.4). One milliliter each of the fresh liquid formulations (equivalent to 10 mg drug) was filled into the dialysis bag which was closed from both ends using two clamps. Each bag was placed in a container filled with 50 mL of the release medium (PBS, pH = 7.4) and shacked at a rate of 100 rpm in a shaker incubator (IKA KS 4000, Germany) adjusted at 37 °C ± 0.5 °C. Samples of 1 mL were withdrawn at predetermined time intervals and replaced with 1 mL of fresh PBS of pH = 7.4. They were measured spectrophotometrically at 251 nm (Shimadzu UV–VIS Spectrophotometer, UV-1800, Japan) after appropriate dilution. Experiments were run in triplicates and the concentration of the drug was calculated from the equation of the developed standard curve of concentration versus absorbance and the time required for 80% of the drug to be released (t_80%_) was calculated after fitting the release profile to the appropriate kinetic equation.

##### Optimization of the prepared in situ forming composite hydrogel systems using desirability function

The desirability function is used to select the formulation(s) with the most suitable characteristics among other formulations based on mathematical equations as applied and described in details in previous studies [32, 33]. The chosen characteristics to be involved in the desirability study were the in vitro gelation time, the viscosity of the fresh liquid mixtures, the viscosity of gels, and the time required to release 80% of the drug from the hydrogels (t_80%_). These responses were ranked between 0 and 1 and the targeted criteria were set as maximizing the gelation time, and viscosity of the gel while minimizing the viscosity of the fresh liquid mixture. The formulation (s) having the highest rank among the tested formulations was selected for further investigations.

#### Preparation of spray-dried linezolid-loaded nanoparticles

Aiming to achieve more sustainment effect on the release profile of the drug, linezolid was encapsulated in polymeric nanoparticles before incorporation into the in situ forming hydrogel. Nanoparticles were prepared via the spray-drying technique (Büchi^®^ nano-spray dryer B-90, Büchi Labortechnik, Switzerland) using PVP K30 or cetyl alcohol as carriers (wall materials) and poly(dl-lactic-co-glycolic) acid and poly (dl-lactide) as release-controlling polymers. A preliminary study was performed to adjust the operation parameters of the apparatus namely the ratio of drug to carrier, the size of spraying nozzle, the inlet and outlet temperatures, and the nitrogen flow rate during the preparation. Briefly, accurate amounts of linezolid and the carriers (PVP K30 or cetyl alcohol) in ratios of 1:1 or 1:5 w/w were dissolved in 20 mL of suitable solvent (acetonitrile or acetone, respectively) using bath sonicator (Elmasonic S30H, Germany) until clear solutions were obtained. After reaching the optimum operation condition, the release-controlling polymers (poly-dl-lactide/glycolide 75:25, 85:15 copolymers and poly-dl-lactide polymer), were dissolved in the drug carrier solutions in ratio of 1:5 w/w using sonicator for 5 min and the prepared solutions were sprayed applying the optimized operation conditions (nano spray dryer nozzle of 7.0-μm size, inlet temperature of 85 °C, outlet temperature of 40 °C, and the flow rate of nitrogen was set at 110 L/min in a closed mode configuration). Drying of nanoparticles was carried out using nitrogen gas that was monitored for oxygen content of less than 4%, by the inert loop B-295 system of the dryer. The dried particles were collected from the inner wall of the electrostatic cylinder electrode of the dryer with the aid of a scraper. The compositions of the prepared nanoparticles are presented in Table 2.

**Table 2:** The composition and physicochemical characterization of the nano-spray-dried linezolid loaded nanoparticles (Data are mean ± SD, n = 3)

Formula	Linezolid (mg)	Carrier (mg)		Polymer (mg)			Yield (%)	Drug content (%)	Particle size (nm)	PDI	Zeta potential (mV)	t_80%_ (hr)	Desirability	
		PVP K30	Cetyl alcohol	P75	P85	P100							Maximized PS	Minimized PS
NP1	100	500	**–**	500	–	**–**	56.30 ± 2.55	97.30 ± 1.27	314.2 ± 3.11	0.342 ± 0.03	-19.45 ± 0.91	26.15 ± 0.39	0.00	0.00
NP2	100	500	**–**	**–**	500	**–**	65.70 ± 0.42	99.22 ± 0.11	382 ± 27.86	0.374 ± 0.02	- 19.75 ± 0.21	39.25 ± 4.13	0.14	0.37
NP3	100	500	**–**	**–**	**–**	500	74.25 ± 3.18	99.70 ± 0.28	390.25 ± 16.62	0.346 ± 0.04	-19.75 ± 0.49	45.25 ± 3.81	0.21	0.853
NP4	100	**–**	500	500	**–**	**–**	73.00 ± 4.24	102.00 ± 1.41	1242 ± 31.11	0.64 ± 0.02	-17.1 ± 0.84	39.57 ± 1.58	0.41	0.30
NP5	100	**–**	500	**–**	500	**–**	76.25 ± 1.06	100.40 ± 0.71	1452.5 ± 24.75	0.596 ± 0.004	-18.75 ± 1.48	83.16 ± 1.49	0.76	0.39
NP6	100	**–**	500	**–**	**–**	500	78.50 ± 2.12	101.90 ± 0.14	1604 ± 26.87	0.584 ± 0.02	-14.15 ± 2.33	130.67 ± 0.14	1.00	0.00

*P75* PLGA with L/G ratio of 75:25 copolymer, *P85* PLGA with L/G ratio of 85:15 copolymer, *P100* Poly-DL-lactide polymer

#### Evaluation of the spray-dried linezolid-loaded nanoparticles

##### Determination of the percentage yield

The collected spray-dried nanoparticles were weighed and the percentage yield (%yield) was calculated according to the following equation:

2 $$\mathrm{\%\,Yield=}\frac{Wf}{Wi} x 100$$

where *W*_*f*_ is the final weight of the collected particles from the nano spray dryer and *W*_*i*_ is the weight of the initial solid contents (sum of the weight of drug, carrier, and polymer before spraying).

##### Determination of percentage drug content

Specific weights of the collected spray-dried nanoparticles (equivalent to 2 mg of linezolid) were dissolved in 12 mL acetonitrile using bath sonicator. The solution was diluted with acetonitrile and measured for linezolid content at wavelength of 259 nm (Shimadzu UV–VIS Spectrophotometer, UV-1800, Japan). The percentage drug content was calculated according to the following equation:

3 $$\mathrm{\%\;Drug\;content=}\frac{\mathrm{Actual \;drug \;content}}{\mathrm{Theoretical\; drug\; content}}\times100$$

##### Measurement of particle size, polydispersity index (PDI), and Zeta potential

Ten milligrams of the spray-dried particles was dispersed in 1 mL distilled water using sonicator for 30 s. The dispersion was further diluted with 14 mL distilled water and were evaluated for their particle size, PDI, and zeta potential using Zeta sizer (Malvern-ZEN^®^ 3600, Nano-series Instrument, Worcestershire, UK) at temperature of 25 °C.

##### In vitro release study of linezolid from the spray-dried nanoparticles

The in vitro release of linezolid from the spray-dried linezolid-loaded nanoparticles (equivalent to 10 mg of the drug) was carried out using the dialysis cellulose membrane diffusion technique as previously described in the “In vitro release of linezolid from the composite hydrogel systems” section. After fitting the release profile to the appropriate kinetic equation, the t_80%_ of the drug release was calculated.

##### Effect of storage on the physicochemical properties of the spray-dried linezolid nanoparticles

Nanoparticles under investigation were sealed in glass vials and stored at both ambient temperature (25 to 30 °C) and refrigerator temperature (2–8 °C) for 3 months. They were evaluated for changes in the percentage drug content, particle size, PDI, and zeta potential as described previously.

##### Optimization of the spray-dried linezolid-loaded nanoparticles

The nanoparticles with appropriate characteristics were selected after applying the desirability function equations, adopting the criteria of maximizing the percentage of yield and the time required for 80% release. The process was conducted twice once to maximize the particle size and the other to minimize it. The nanoparticles having the highest desirability function in both cases were selected for incorporation in the optimized composite hydrogel system.

##### Scanning electron microscopy (SEM)

The optimized spray-dried linezolid nanoparticles were examined for their shape and surface morphology using a scanning electron microscope (SEM; JSM-6400; JEOL Ltd., Tokyo, Japan). The samples were coated with thin layer of gold and examined at a voltage of 20 kV.

##### Fourier transform infrared spectroscopy (FT-IR)

FT-IR of the selected formulations, their individual components, and physical mixtures was carried out to detect any possible chemical interactions between components of the selected formulations. A quantity of 2 mg of each investigated item was mixed with dry potassium bromide and the mixture was compressed into a disc. The disk was scanned over a range of wave lengths from 4000 to 650 cm^−1^ using FT-IR apparatus (Agilent CARY 630, USA).

##### Differential scanning calorimetry (DSC)

DSC analysis was performed by precisely weighing 2 mg of each of the selected spray-dried systems, their individual components, and physical mixtures. Samples were placed in a hermetically sealed aluminum pans, which were heated at a constant rate of 10 °C/min under nitrogen atmosphere from 25 to 350 °C. The DSC testing was carried out using Shimadzu differential scanning calorimetry (DSC-50; Shimadzu, Kyoto, Japan).

#### Preparation of the in situ forming hydrogel/nanoparticle-combined systems

Accurately weighed (220 mg) of the selected spray-dried nanoparticles (NP3 and NP6, equivalent to 20 mg linezolid) and 20 mg nHA were dispersed into 1 mL of chitosan solution on a magnetic stirrer to obtain a homogeneous mixture. An equal volume (1 mL) of NaHCO_3_ as a gelling agent was added portion wise while stirring to the chitosan solution to obtain a mixture having linezolid concentration of 10 mg/mL.

#### Evaluation of the in situ forming hydrogel/nanoparticle-combined systems

The rheological properties, gelation time, and in vitro drug release of the combined systems were investigated as described previously under the composite hydrogel and SEM of the lyophilized hydrogels were examined as described under nanoparticles.

The injectability of the combined systems was assessed using home-developed instrument to simulate the one described in literature [35] with some modifications. One milliliter of the hydrogel was placed into a 19-gauge hypodermic syringe of 3-mL capacity. The hydrogel-filled syringe was then affixed to a rubber tube end, which was connected to an air pump. In order to determine the injectability, the air was forced from the air pump onto the hydrogel surface. A sphygmometer was used to keep the pressure on the hydrogel surface constant (at 40 mmHg). To determine the injectability of the formulations, the values of the flow rate (mL/s) can be estimated and compared by recording the time it takes to expel the 1-mL sample. The prepared composite hydrogel formulations were compared to an oily commercial injection (Betolvex^®^) to assess their suitability for injection.

The pH of the combined systems was measured before and after the addition of NaHCO3 solution in order to assess the effect of its addition on the pH of the mixture and the suitability of the final system for injection. The pH was measured using a calibrated digital pH meter (JENWAY 3505, UK) at 25 °C. The electrode was exposed to the chitosan dispersion containing the nanoparticles before and after the addition of NaHCO_3_ until a constant reading was recorded on the screen [36].

To assess the stability of the hydrogel/nanoparticle-combined system, they were sealed in glass vials and stored at both ambient temperature (25 °C ± 3 °C) for a month (a preliminary study) and refrigerator temperature (5 °C ± 3 °C) for 3 months. Combined systems were stored in refrigerator in two forms: (1) the gelling agent; NaHCO_3_ solution was mixed with the chitosan solution containing nanoparticles and nHA in a readymade form before storage and (2) both solutions were mixed after storage immediately before evaluation. At the end of the storage period, they were assessed for their physical appearance, viscosity, in vitro gelation time, in vitro drug release, and pH.

#### Gamma sterilization

The selected combined hydrogel/nanoparticle systems for in vivo study were subjected to gamma sterilization. The chitosan dispersion containing drug nanoparticles and nHA as well as a 2% NaHCO_3_ solution were separately subjected to sterilization using gamma irradiator at 10 kGy (1.88 kGy/h) (Gamma cell 1000; BEST Theratronics, Ontario, Canada). During the gamma sterilization process, the products were placed inside containers made of polyurethane pre-filled with dry ice to prevent melting or aggregation of particles [37]. After the sterilization process, the two components of the systems were mixed as described under the “Preparation of the in situ forming hydrogel/nanoparticle-combined systems” section to obtain the combined hydrogel/nanoparticle system and were re-tested for viscosity, drug content, in vitro gelation time, in vitro drug release, and pH.

#### In vivo evaluation of selected systems

The protocol of the study was conducted according to the ethics guidelines and regulations of the international guiding principles for biomedical research involving the use of animals after being approved by the Research Ethics Committee of Faculty of Pharmacy, Cairo University, Egypt (REC, PI 2081, valid since 26/09/2022).

##### Animals

Sixteen male New Zealand white rabbits, of 8 to 12 weeks old and 2.5 to 3.5 kg body weight, were enrolled in this study. Rabbits were examined carefully to make sure they are not suffering from any skeletal or general health conditions. They were left for a week for acclimatization while fed on commercial balanced diet and water was ad libitum.

##### Infectious bacterial strain

Methicillin-resistant *Staphylococcus aureus* (MRSA) strain used in this study was isolated from a systemic infection. The linezolid minimum inhibitory concentrations (MICs) to the clinically isolated MRSA was found to be 3.25 μg/mL. MRSA strain was grown for 16 h at 37 °C in tryptic soy broth and bacterial concentrations were estimated spectrophotometrically, where an A600 of 1.0 corresponds to 6.6 × 10^8 colony-forming units (CFUs)/mL, as determined by a separate standard curve relating the A600 to CFU. Briefly, 1 mL of a 16-h bacterial culture was collected, centrifuged (at 5000 rpm for 3 min), the supernatant was discarded and the pellet was re-suspended in 1 mL of phosphate buffer saline (PBS), from which 50 μL was removed and diluted with 950 μL of PBS. The overnight bacterial culture was adjusted using PBS, and 0.1 mL of 1 × 10^9 CFUs of MRSA/mL was used in the experiment.

##### Study design and experimental procedure

Rabbits were randomly arranged into 4 groups each having 4 rabbits namely a control group receiving no treatment, group I receiving composite hydrogel system, H1, groups II and III receiving combined hydrogel/nanoparticle system: H1NP3 and H1NP6, respectively. Rabbits were generally anesthetized using Xylazine HCl (3 mg/kg) and ketamine HCl (10 mg/kg) injected intramuscularly. The right hind limbs were prepared aseptically focusing on the proximal tibial region medially. A bone marrow biopsy needle was introduced percutaneously through the medial aspect of the right tibial metaphysis reaching the intramedullary cavity, where 0.1 mL of infectious bacteria (MRSA) in sterile saline were injected for the induction of bone infection. Animals were left for 4 weeks post-injection of bacterial solution afterwards groups I, II, and III were injected with 1 mL of the treatment formulae namely H1, H1NP3 and H1NP6, respectively each equivalent to 10 mg of linezolid. Injections were performed under general anesthesia using 18-G hypodermic syringe in the area between the tibial shaft and posterior tibial muscle.

##### Post-operative evaluations

All animals were inspected daily at the injected limb and the treatment site. Rabbits were subjected to weighing via digital balance and recording of their rectal temperature every 3 days. Under general anesthesia, plain radiographs of right tibias were taken at 2, 4, and 8 weeks post-infection.

Ear vein blood samples were withdrawn almost every week during both of the infection induction and the treatment periods as well as before induction of infection (as a baseline) and subjected to both blood hematology and biochemistry studies. For hematology studies, they were inspected for erythrocyte sedimentation rates (ESRs) using the DISPETTE-2-kit (Fisher Scientific, Cambridge, Massachusetts, USA) within 24 h of blood collection, C-reactive protein (C-RP) levels using CRP latex agglutination (SPINREACT, Spain), and white blood cells (WBCs) count using ProCyte Dx Hematology Analyzer (IDEXX, USA). For biochemistry studies, a biochemical analyzer (MicroLab, India, 2022) was used, where blood serum parameters were determined applying a standardized method; alkaline phosphatase (ALP)—adapting a unified method with nitrophenyl phosphate using a commercial ALP Kit (Chema Diagnostics, Italy) and serum total Ca & P were determined according to manufacturer’s instructions.

##### Euthanasia

Animals were euthanized using thiopental sodium 100 mg/kg by intraperitoneal (i.p.) injection. Animals of the control group were euthanized at 2, 4, and 8 weeks post-infection while those of the treatment groups were euthanized at 8 weeks post-infection (4 weeks post-treatment).

##### Histopathological evaluation

The right tibias were harvested after animals’ euthanasia and the bone specimens were fixed in 10% neutral buffer formalin, decalcified by 10% formic acid, trimmed, washed with water, dehydrated in ascending grades of ethyl alcohol, cleared in xylene, and embedded in pre-heated paraffin. A section of 4–6 μ was processed, stained with hematoxylin and eosin stain [38], and examined under light microscope (CX21 Olympus microscope, Tokyo, Japan).

#### Statistical analysis

All experiments were carried out in triplicate and results were expressed as mean ± SD. The significance of the effect of factors were tested using one-way ANOVA using SPSS software (SPSS statistics 17.0, USA). Significance differences between results were considered when *p* < 0.05.

## Results and discussion

### Preparation of in situ forming composite hydrogel of chitosan loaded with linezolid and nanohydroxyapatite

While chitosan is only soluble in acidic medium, the addition of a weak base such as GP or NaHCO_3_ leads to the formation of a hydrogel-like precipitate upon increasing the pH above 6.2 (the pKa of chitosan) only at 37 °C [18]. In the current study, three different concentrations of GP (25%, 50%, and 75%) and NaHCO_3_ (1%, 2%, and 4%) were used for the preparation of in situ forming hydrogel. However, the additional of 25% of GP and 1% of NaHCO_3_ solutions in ratios of 1:9 v/v and 1:1v/v, respectively to the 2% chitosan solutions failed to affect gelation over a period of two hours at 37 °C both in absence and presence of nHA (which was found to significantly reduce the gelation time for both gelling agents). This could be due to the incomplete neutralization and cross-linking of the 2% chitosan solution in the presence of such low concentrations of both gelling agents, hence were excluded from farther investigations. Gelation was successful at 50% and 75% of GP solutions at a ratio of 1:9 v/v of GP solution:2% chitosan solution and 2% and 4% of NaHCO_3_ at a ratio of 1:1 v:v of NaHCO_3_:2% chitosan solution. It is worth noting that nHA reduced the gelation time to one-half the original period of time for the GP solutions and to one 4th the original period in case of NaHCO_3_ solutions (data are not presented).

### Evaluation of the prepared in situ forming hydrogel systems

#### Effect of the concentration of gelling agent on the in vitro gelation time

The gelation time of selected formulations (H1–H4) ranged from 3.5 to 6.5 min (Table 1) which were considered suitable to keep the mixture in liquid form for injection after mixing the two solutions (to be suitable for injection in cases of ambient temperature above 25 °C). In the same time, this period is short enough to allow fast gelation after injection to the body and prevent the diffusion of the solution away from the injection site in the close vicinity to the infected bone. These results are in correlation with previously published data for injectable in situ forming hydrogel systems [39, 40].

Increasing the concentration of both GP and NaHCO_3_ solutions significantly (*p* < 0.05) reduced the gelation time due to faster deprotonation of the quaternary amine grope of chitosan acidified solution at higher concentration of the gelling agent and the precipitation of chitosan in cross-linked three-dimensional structure entrapping massive amount of water within its network structure, and hence affecting faster gel formation [41]. These results are in accordance with previously published data for both systems [17, 19, 42].

#### Rheological properties

As some formulations may be too viscous to enable injectability, the viscosity of hydrogel formulations was tested at ambient temperature (25 °C, corresponding to the temperature of mixing the two solutions and administration) and at 37 °C (corresponding to the body temperature at which gel formation occurs). Viscosity was measured throughout a range of 10 rpm, corresponding to the product at rest condition and 250 rpm, corresponding to the product under syringebility across the needle of the syringe (with 10-rpm increment every 1 min). Results revealed that upon increasing the shear rate, viscosity decreased significantly to one-fourth its original values for all formulations (H1–H4), indicating a shear thinning behavior which is advantageous for injectable systems (Table 1). Furthermore, applying these results to Farrow’s equation revealed *R*^2^ values of 0.9939, 0.9953, 0.9947, and 0.9932 for formulations H1, H2, H3, and H4, respectively and Farrow’s constant (*N*) of 1.609, 1.63, 1.874, and 1.898, respectively which confirm a non-Newtonian pseudoplastic flow characteristics to the hydrogel systems.

The fresh mixed formulations prepared with GP as gelling agent at ambient temperature was found to be significantly (*p* ˂ 0.05) higher at both high and low shear rate than those prepared with NaHCO_3_. The higher viscosity may be ascribed to the higher ratio of chitosan solution:GP solution (9:1) compared to chitosan:NaHCO_3_ solutions (1:1) which reflect lower dilution of the viscous dispersion of chitosan in the first case compared to the second one. Conversely, the viscosity of the formed gel at 37 °C for formulations prepared with NaHCO_3_ was three- to fivefolds higher than those prepared with GP as gelling agent. This may be due to the low mechanical strength demonstrated by scaffolds of chitosan prepared using GP as a gelling agent as previously presented by Deng et al. [43] who reported the enhancement of their mechanical strength upon addition of NaHCO_3_ to the formed hydrogel.

Increasing the concentration of the gelling agents did not have significant effect on the viscosity of the freshly mixed formulation in liquid forms, both at low and high shear rates; however, it significantly increased the viscosity of the formed gel at 37 °C. This effect may be attributed to the higher degree of deprotonation of the quaternary amine group of chitosan by the gelling agent at the higher concentration leading to more polymer precipitation, cross-linking, and entrapment of liquids (gelation). It is worth noting that this deprotonation effect of the gelling agent is only significant at the gelling temperature of 37 °C. Similar results were reported by Kempe et al. [44] who observed an increase in the gel viscosity upon doubling GP concertation from 8 to 16% and by Abdel-Salam et al. [17], who reported that the effect of NaHCO3 concentration is only influential at 37 °C.

#### In vitro release of linezolid from the in situ forming hydrogel

**Fig. 1:**
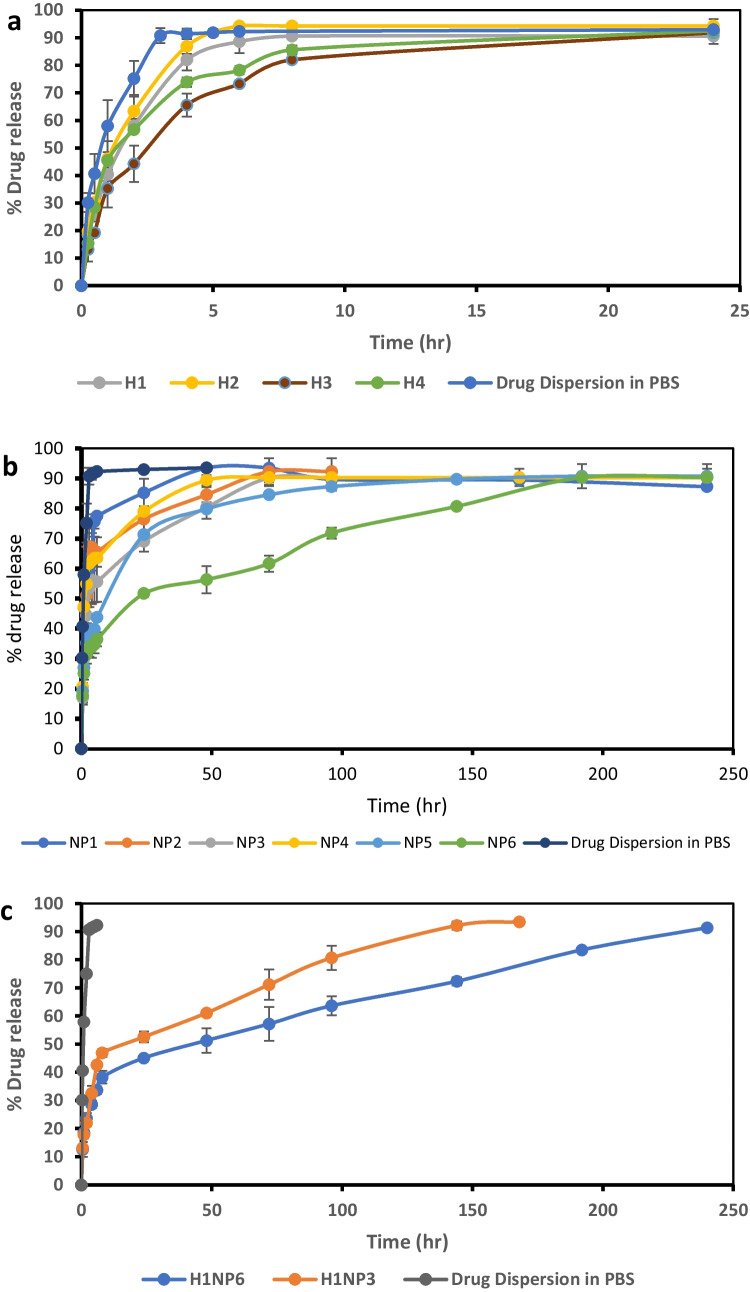
Release profile of linezolid in phosphate buffer saline (PBS) of pH = 7.4 from: **a** In-situ forming hydrogels, **b** linezolid-loaded , spray-dried polymeric nanoparticles and **c** combined In-situ forming hydrogel/nanoparticle systems in comparison to free drug dispersion in PBS of pH 7.4

The release profile of linezolid from the chitosan in situ composite hydrogels (H1–H4) are presented in Fig. 1a. The obtained release data were fitted to various kinetics model equations (zero, first orders, and Higuchi diffusion model). The four formulations of the hydrogel were found to follow Higuchi diffusion release kinetics. The release data were further fitted to Korsmeyer-Peppas equation [45] where the coefficient of release, *n* was found to be 0.386, 0.4285, 0.4123, and 0.4521 for H1, H2, H3, and H4, respectively, confirming a Fickian diffusion of linezolid from the composite hydrogels. The time required for the release of 80% of the drug contents was calculated from the best fitted equation (Table 1).

The release profile of the hydrogel showed a burst release ranging from 15 to 20% in the first few minutes which can be justified on the basis of the time required for developing the gel at the release medium at 37 °C which ranges from 3.5 to 6 min as discussed previously. The burst effect from the drug dispersion in phosphate buffer saline of pH 7.4 was significantly higher (*p* ˂ 0.5) exceeding 40%. After the initial burst release, the drug was trapped inside the developed hydrogel and hence was released slowly over a period of 8 to 19 h in comparison to 3.5 h from the drug dispersion.

The time required for 80% release of linezolid was two-folds longer for the hydrogels prepared with NaHCO_3_ as gelling agent (8–9 h) compared to those prepared with GP (4–5 h). These results correlate with the higher viscosity of the developed hydrogel with NaHCO_3_ compared to those developed with GP, which exert more retarding effect towards the penetration of the release medium to extract the drug and/or the diffusion of the drug molecules out of the congealed matrix. It could be also due to the higher mechanical strengths of the former hydrogel compared to the latter one as reported previously [43], which act against the erosion of the hydrogel and prevent surface area expansion and hence limit the rate of release. However, no significant difference (*p* ˃ 0.5) was observed in the t_80%_ upon increasing the concentration of either of the two gelling agents.

As a conclusion, linezolid was released from the composite hydrogels over a period of 24 h. These hydrogels may release the drug in much longer period upon injection in the close vicinity of bone tissues with the limited volume of interstitial fluids compared to the volume of dissolution medium in addition to being injectable which allow frequent administration. Nevertheless, a more sustained release pattern of the drug was thought to be required to reduce the frequency of injection and increase patinates’ compliance since the treatment of acute osteomyelitis would require a period of 4–8 weeks [46] and the chronic one would require much longer period. Therefore, an optimized composite in situ forming hydrogel can act as a vehicle to a more sustained release form of the drug, namely polymeric encapsulated one instead of the free form.

#### Optimization of the prepared in situ forming composite hydrogel using desirability function

For selecting the gel system of appropriate characteristics, the desirability function equations were applied adopting the following criteria: (1) minimizing the viscosity of the fresh compounded liquid system to facilitate injection; (2) maximizing the gelation time to allow suitable period for injection after compounding chitosan solution with the gelling agent solution specially at elevated ambient temperature; (3) maximizing the viscosity of the formed gel at 37 °C and the time required for 80% release to guarantee sustainment of the drug release from the gel matrix. Results revealed that H1 system prepared with 2% chitosan solution containing 10 mg/mL of each of linezolid and nHA compounded with 2% NaHCO_3_ solution in a ratio of 1:1 of chitosan solution:NaHCO_3_ solution had the maximum desirability value of 0.7244 (Table 1), therefore, was selected to incorporate a more sustained release form of the drug.

### Preparation of spray-dried linezolid nanoparticles

Nano Spray Dryer-B90 was successfully employed in earlier studies to produce particles in the nano to micro size range with a high production yield [47–50]. A preliminary study for adjusting the operation parameters of the apparatus using drug solution containing two types of carriers namely PVP K30 (a water-soluble carrier) and cetyl alcohol (a lipid soluble one) in ratios of 1:1 and 1: 5 of drug:carrier each was conducted. Results revealed that spray drying at 1:1 drug to carrier ratio produced much lower yield than the 1:5 ratio which could be attributed to sticking the drug to the inner cylinder of the drying chamber as a result of the insufficient amount of carrier to protect the drug at such low ratio. It is worthy to note that particle loss from the spray-drying process can occur because of several factors including deposition on the drying chamber wall, escape of fine particles from the outlet of air filter and powder loss during powder collection [51]. Therefore, carrier ratio to drug is considered a critical factor in the spray-drying process as it significantly impacts the % yield [52–54].

Concerning the particle size, results revealed that upon using PVP K30 as a carrier, increasing the drug:carrier ratio from 1:1 to 1:5 resulted in significantly smaller particle size (*p* < 0.05). This may be due to the hydrophilic nature of PVP K30 which facilitate the reduction of surface tension of the formed droplets during spring and hence facilitate their sub-deviation and prevent their coalescence during drying. These results are in accordance with those reported by [47]. On the other hand, increasing the ratio of cetyl alcohol:drug from 1:1 to 1:5 resulted in significantly larger particle size. This can be explained on the basis that the presence of high amount of the lipophilic carrier (cetyl alcohol) in the aqueous system had self-associating characteristics as a result of hydrophobic interactions, which may result in enlarging the particle size as reported previously [55]. The preliminary study also revealed that a nano-spray dryer nozzle of 7.0-μm size, an inlet temperature of 85 °C, outlet temperature of 40 °C, and the flow rate of nitrogen to be set at 110 L/min were appropriate running conditions for the specified system.

### Evaluation of the spray-dried linezolid nanoparticles prepared under the optimized operation conditions

#### Effect of polymer and carrier types on the percentage yield

Upon addition of PLGA or PLA polymers to the solution containing the drug and carriers in ratio of 1:5 drug to carriers, according to the described method, a significant increase (*p* < 0.05) in the % yield was observed in comparison to the formulation lacking polymers. A probable explanation is that increasing the solid content in the feed solution could result in supporting the production of particles, thus reducing the percentage loss from the total weight of the patch. Similar results were reported previously [56].

Applying ANOVA revealed a significant effect of both the type of carrier and polymer on the % yield. Upon changing the carrier type from PVP K30 (NP 1–3) to cetyl alcohol (NP 4–6) a significant increase in the % yield (*p* = 0.0004) was recorded (Table 2). This could be attributed to the ability of PVP 30 to reduce the particle size and hence increase the fraction of escaped fine particles from the outlet of air filter. It could also be due to the sticky nature of PVP K30 upon drying which results in partial adherence of particles to the wall of the drying chamber causing loss of the recovered particles as reported previously [57–59]. On the other hand, cetyl alcohol being lipophilic in nature has lower tendency to adhere to the wall of the drying chamber which opposes the reduction in the % yield.

Regarding the type of polymer, it was found that increasing the lactide to glycolide ratio (L/G) in the copolymer composition from 75/25 to 85/15 or further using a pure poly-lactide polymer resulted in increasing the % yield significantly (*p* = 0.002), which was in agreement with the results obtained by [47]. This could be ascribed to the fact that increasing the lactide in a polymer composition is accompanied by increasing its glass transition temperature which reduces the particle stickiness and/or agglomeration within the spray drier [47]. As presented in Table 2, it is clearly evident that spray-dried linezolid nanoparticles prepared using poly-lactide polymer and drug to cetyl alcohol ratio of 1:5 (NP6) exhibited the highest % yield among all the prepared formulae (78.5%).

#### Determination of the percentage drug content

As presented in Table 2, the % drug content of the tested nanoparticle batches ranged between 97.30 ± 1.27 and 102.00 ± 1.41, indicating the complete encapsulation of the drug within the polymeric particles and the minimum loss if any. The low level of variability as reflected by the small standard deviation indicates the homogeneity of distribution of the drug among the particles, confirming the suitability of the selected parameters of the formulation and the suitability of the spray-drying technique as encapsulating tool of the drug under the specified conditions.

#### Effect of polymer and carrier types on particle size, PDI, and zeta potential

The physicochemical characteristics of the polymeric nanoparticles of linezolid are presented in Table 2. It was observed that the particle size varied between 314.2 (NP1) to 1604 nm (NP6). ANOVA revealed that both the type of carrier and the type of polymer significantly (*p* ˂ 0.05) affected the particle size. The introduction of a lipid material (cetyl alcohol) into the solution resulted in significantly larger particle size in comparison to formulations prepared using hydrophilic material (PVP K30) as carrier (*p* < 0.0001). Similar results were reported previously [60] during the preparation of liposomes using cetyl alcohol as a lipophilic component, where larger particle size was obtained using cetyl alcohol compared to the corresponding systems lacking it. Another study revealed that increasing the amount of cetyl alcohol had significantly positive effect on the particle size during the preparation of elastic rosuvastatin calcium nanovesicles [61].

Regarding polymer type, it was observed that the particle size was also significantly affected by varying L/G ratio in the polymer chain (*p* < 0.0001). Increasing the (L/G) ratio from 75:25 (NP1 and FNP4) to 85:15 (NP2 and NP5) and then to the pure poly lactide (NP3 and NP6) resulted in significant increase in particle size both in case of PVP K30 and cetyl alcohol as carriers. This may be due to the increased hydrophobicity of the polymer with increasing the lactide moiety leading to higher tendency for agglomeration in the aqueous system [47, 55].

PDI value is measured to detect the uniformity of particle size of the prepared products, where PDI values < 0.5 reflect uniform particle size distribution [62]. The PDI values of the prepared spray-dried linezolid particles ranged from 0.342 to 0.64. Significant increase (*p* < 0.0001) in PDI values was observed in particles prepared using the hydrophobic carrier cetyl alcohol (NP 4–6) compared to those prepared with the hydrophilic carrier (PVP K 30) (NP 1–3). Higher PDI values of the formulations along with larger particle sizes confirms the aggregation of particles upon using the lipid carrier in the aqueous system. However, increasing the (L/G) ratio in the copolymer chain or changing to pure poly-lactide did not significantly (*p* = 0.5047) change the PDI for both carriers.

Zeta potential detect the physical stability of particles through determination of the surface charge of nanoparticles in a colloidal dispersion [63]. Zeta potential of the prepared formulations ranged from − 13.85 to − 19.45 mV. These surface charge confirms the stability of the prepared linezolid spray-dried nanoparticles. Statistical analysis revealed a significant increase in zeta potential among systems containing PVP K30 as a carrier compared to those prepared using cetyl alcohol (*p* = 0.0118). This may be attributed to the increased oxygen atoms in presence of PVP K30 on the particles, which contributes to large electron cloud responsible for negative charges [64].

#### In vitro release of linezolid from spray-dried nanoparticles

In vitro release study was carried out for the formulated nanoparticles; NP1-NP6 and the data were fitted to various kinetics release models (zero order, first order, and Higuchi diffusion). All 6 nanoparticles followed Higuchi diffusion release kinetics which was further confirmed by fitting the data to Korsmeyer-Peppas equation [45]. The time required for releasing 80% of the drug was calculated from the diffusion equation as presented in Table 2. The *n* values were 0.3515, 0.302, 0.4677, 0.328, 0.290, and 0.251 for NP1 to 6, respectively, thus confirming the Fickian diffusion nature of linezolid from the spray-dried nanoparticles.

The release profiles from different nanoparticles in comparison to that from the drug dispersion in PBS of pH = 7.4 are illustrated in Fig. 1b. An initial burst release of the drug ranging from 10.69 ± 2.33% (NP6) to 19.19 ± 0.175 (NP1) was observed, which may be due to the presence of some unproperly coated drug crystals close to the surface of the spray-dried nanoparticles which dissolve fast to the release medium. Compared to the release from the free drug dispersion, all spray-dried nanoparticles dispersions showed significantly lower (*p* < 0.05 burst effect reaching one-half to one-quarter of that from the free drug dispersion (40%).

Both carrier and polymer types had significant effect on the release rate of the drug as reflected by the time required for 80% release. Changing the carrier type showed t_80%_ ranging between 26.15 to 45.25 h for PVP and 39.57 to 130.67 h for cetyl alcohol (significance: *p* < 0.0001). The difference in the sustainment effect is attributed to the hydrophobic nature of cetyl alcohol which isolate the drug particles from being in direct contact with the aqueous release medium for longer period of time in comparison to the hydrophilic nature of PVP K30 that can improve the dissolution efficiency of the drug through its wetting effect. Similarly, it was reported that using PVP as a carrier in the spray drying of a solid dispersion of bicalutamide reduced its mean dissolution time and improved its dissolution efficacy [65]. Additionally, PVP K30 can act as a pore forming agent where upon its dissolution it creates pours particles that facilitate drug release as a result of increasing the release surface aria. This finding is in agreement with the results obtained by Park, S.C., et al. [66] who investigated the effect of adding PVP as hydrophilic additive to the hydrophobic poly-lactic acid matrix. A similar finding was also reported previously [67] upon incorporating PVP as pore forming agent into PLGA microparticles.

Regarding the effect of lactide to glycolide (L/G) ratios in the copolymer chain (75:25, 85:15) as well as the use of pure poly-lactide polymer, they significantly prolonged the time required for 80% release (*p* < 0.0001). The reason is referred to the fact that increasing the lactide moiety reduces the hydrophilicity of the polymer and hence repels the dissolution medium from coming in direct contact with the particles for longer period of time. These results correlate with those reported previously [29, 68] which investigated the effect of different ratios of (L/G) in polymer and found out that increasing the lactide ratio, significantly reduced the percentage of drug release from the formulations.

#### Effect of storage on the physicochemical properties of the spray-dried linezolid nanoparticles

The spray-dried linezolid nanoparticles stored in refrigerator as well as at ambient temperature for 3 months showed no significant differences (*p* > 0.05) in % drug content, particle size, PDI, and zeta potential in comparison to freshly prepared patches (data are not shown) reflecting adequate stability of the solid product.

#### Optimization of the spray-dried linezolid-loaded nanoparticles

Applying the desirability function equations adopting the decided criteria as mentioned previously revealed that while maximizing the particle size, linezolid-loaded spray-dried poly-lactide particles prepared using linezolid:cetyl alcohol:PLA in weight ratios of 1:5:5 (NP6) were the particles of highest desirability function (≈ 1). After adjusting the same criteria with minimizing the particle size, those particles prepared using linezolid:PVP K30:PLA in weight ratios of 1:5:5 (NP3) were the one with the highest desirability function (0.853). The two selected nanoparticles were subjected to further investigations.

#### Scanning electron microscopy (SEM)

**Fig. 2:**
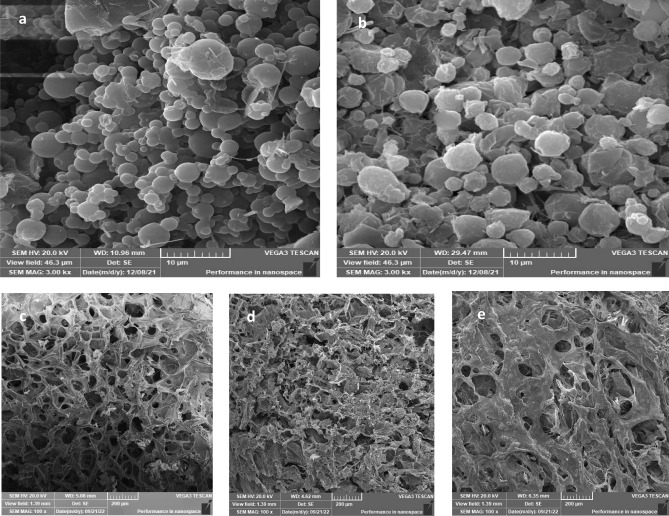
Scanning electron microscopy of **a** nanoparticles NP3, **b** nanoparticles NP6, **c** Lyophilized composite in situ forming hydrogel system H1, **d** lyophilized in situ forming hydrogel/nanoparticles combined system H1NP3, and **e** Lyophilized in situ forming hydrogel/nanoparticles combined system H1NP6

SEM examination can give insights on the shape and surface properties of the formed nanoparticles. In Fig. 2a, the nanoparticles, NP3, shows spherical particles with smooth surface while these of NP6 (Fig. 2b) show somewhat spherical shape with surface roughness and slight wrinkles, which may be due to the increased content of cetyl alcohol that can form irregular shape when separate during drying and precipitating on the surface of the particles. Similarly, it was observed that the use of fatty alcohol and its concentration in a dispersion affect the extent of roughness of the surface of microparticles [69]. Additionally, it was reported that the excipients employed in the spraying process determine the surface and shape of the spray-dried particles [62].

#### Fourier transform infrared spectroscopy (FT-IR)

FT-IR spectroscopy is used to determine whether an interaction occurred between two or more components in a formulation. Therefore, FT-IR spectroscopy for the selected nanoparticles namely NP3 and NP6, their individual components as well as the physical mixtures of their individual components were conducted and their spectra are presented in Supplementary-material 1. The spectrum of linezolid (Supplementary 1a) showed a characteristic peak at 3361.11 cm^−1^ corresponding to N–H amide and another peak at 2847.77 cm^−1^ corresponding to –OCH_3_.

Other peaks at 1744.4 cm^−1^ and 1118.2 cm^−1^ are characteristic of C = O and C–O stretching vibrations, respectively. Benzene ring C = C in linezolid structure had characteristic vibration at 1513 cm^−1^. The recorded peaks were in accordance to the reported data [70]. The spectrum of poly-lactide (Supplementary 1b) showed characteristic peaks at 2944.6 cm^−1^ and 1453.7 cm^−1^ corresponding to stretching and bending CH_3_ groups, respectively, in addition to a band at 1744.4 cm^−1^ due to ester group, which matches its reported data [71, 72]. In PVP K30 spectrum (Supplementary 1c), a distinguishable broad band for –OH was observed at 3421.7 cm^−1^ in addition to a stretching vibration at 2952.1 cm^−1^ attributed to asymmetric CH_2_ of pyrrole ring, a bands at 1647.5 cm^−1^ and 1282.2 cm^−1^ corresponding to C = O and C–N, respectively as reported previously [73]. The spectrum of cetyl alcohol (Supplementary 1d) showed a characteristic peak at 3324.8 cm^−1^ corresponding to the stretching of –OH vibration, a peak at 2914.8 cm^−1^ corresponding to the C–H stretching vibration, while that at 1461.1 cm^−1^ is due to –CH_2_ stretching vibration. Additionally, those peaks at 1058.6 cm^−1^ and 723.1 cm^−1^ are attributed to C–O stretching vibration which matches the data reported for cetyl alcohol [74]. The FT-IR spectra of the physical mixtures of the components of both NP3 (Supplementary 1e) and NP6 (Supplementary 1f) and their corresponding nanoparticles; NP3 (Supplementary 1g) and NP6 (Supplementary 1h) showed all the observed peaks of the drug which confirms the absence of any chemical interaction between the drug and the other components of the spray-dried nanoparticles.

#### Differential scanning calorimetry (DSC)

DSC is a potential tool for determining physical characteristics including crystallinity and amorphous nature through the estimation of changes in temperature and energy at phase transitions to give insight about melting and recrystallization behavior of crystalline materials [19]. DSC of the selected formulae (NP3 and NP6), their individual components, and their corresponding physical mixtures was carried out and the thermograms are illustrated in supplementary-material 2. The DSC thermogram of linezolid revealed a characteristic sharp endothermic peak at 180.8 °C corresponding to its melting point and indicated its crystalline nature, which is consistent with the literature [75, 76]. Poly-d,l-Lactide showed endothermic peak at 125 °C corresponding to its melting point [77]. PVP K30 displayed a broad endothermic peak ranging from 20 to 100 °C which is attributed to water loss during heating of a hygroscopic polymer [78, 79]. The thermogram of cetyl alcohol showed a sharp endothermic peak at 54 °C corresponding to its melting point which was close to that reported previously [80]. The thermograms of the physical mixtures of the components of NP3 and NP6 revealed that the sharp characteristic peak of linezolid was reduced in size probably due to dilution with the other components of nanoparticles where it completely disappeared from both nanoparticles; NP3 and NP6 indicating complete transformation of the drug to amorphous form within them as reported previously [81, 82].

### Preparation of the in situ forming hydrogel/nanoparticle-combined systems

Based on the desirability study, NP3 and NP6 were selected to be incorporated into the optimized composite hydrogel system, H1, to form a combined composite in situ gelling/nanoparticulate system H1NP3 and H1NP6, which were subjected to further evaluations.

### Evaluation of the in situ forming hydrogel/nanoparticle-combined systems

#### Rheological properties

The results of the viscosity of the freshly prepared liquid form of the combined systems at 25 °C and the gel form at 37 °C are presented in Table 3. Results revealed that upon increasing the shear rate, the viscosity dropped for both systems: H1NP3 and H1NP6 significantly (*p* < 0.05). Furthermore, fitting the rheograms to Farrow’s equation revealed *R*^2^ of 0.9888 and 0.9867 for H1NP3 and H1NP6, respectively with Furrow’s constant (*N*) of 1.8529 and 1.9137, for the same systems, respectively, which confirm a shear thinning behavior for the in situ forming hydrogel/nanoparticle-combined systems that is suitable for injectable forms.

**Table 3:** Evaluation of the in-situ forming hydrogel/nanoparticles combined systems

Combined systems	Viscosity (cP)						Average flow rate of fresh system (mL.min^−1^)	Gelation time (min.)	T_80%_ (hr)	pH
	Fresh at 25 °C		Stored for 1 months at ambient temperature		Gel at 37 °C					
	10 rpm	250 rpm	10 rpm	250 rpm	10 rpm	250 rpm				
H1NP3	470.75 ± 3.88	130.88 ± 1.95	750.15 ± 7.34	149.22 ± 2.22	9575.14 ± 17.64	174.49 ± 8.06	0.378 ± 0.076	2 ± 0.00	93.89 ± 3.15	6.76 ± 0.04
H1NP6	566.01 ± 8.75	149.28 ± 8.37	903.9 ± 6.5	165.81 ± 3.78	11,120.63 ± 13.25	249.69 ± 10.59	0.187 ± 0.085	2.5 ± 0.14	166.28 ± 3.32	6.92 ± 0.02
Oily injection	-	-			-	-	0.449 ± 0.052	-	-	-

Viscosity of the liquid hydrogel at 25 °C at the lowest shear rate were 470.75 cP ± 3.88 and 566.01 ± 8.75 for H1NP3 and H1NP6, respectively. The combined systems displayed significantly higher (*p* < 0.05) viscosity than the composite hydrogel containing the free drug. This can be attributed to the incorporation of spray-dried linezolid-loaded PLA nanoparticles, which form heavier dispersion being insoluble in the chitosan composite in situ gelling system.

It was observed that the viscosities of both the freshly prepared liquid at 25 °C and the gel formed at 37 °C for H1NP3 system were significantly (*p* < 0.05) lower than those determined for H1NP6 system. This may be due to the presence of the hydrophobic carrier (cetyl alcohol) which imparts larger particle size to the nanoparticles and more tendency for aggregation in the aqueous medium in contrast to the hydrophilic carrier (PVP K30).

#### Injectability study

Since the injectability of a hydrogel system is crucial to be able to deliver it to the infected site, the mean flow time (min) was determined and the mean flow rate (mL/min) was calculated for both system and compared to those of a commercially available oily injection (Table 3). Results revealed that the flow rate of H1NP3 (0.378 ± 0.076) did not differ significantly from that of the commercial oily injection (0.449 ± 0.052). However, a significant difference (*p* < 0.05) was observed between the flow rate of H1NP6 (0.187 ± 0.085), and both of H1NP3 and the oily injection, they were all injectable. The observed difference between the injectability of H1NP6 and H1NP3 is attributed to the significant difference in the recorded viscosity of the two fresh liquid formulations at 25 °C at the same shear rate.

#### In vitro gelation time

**Fig. 3:**
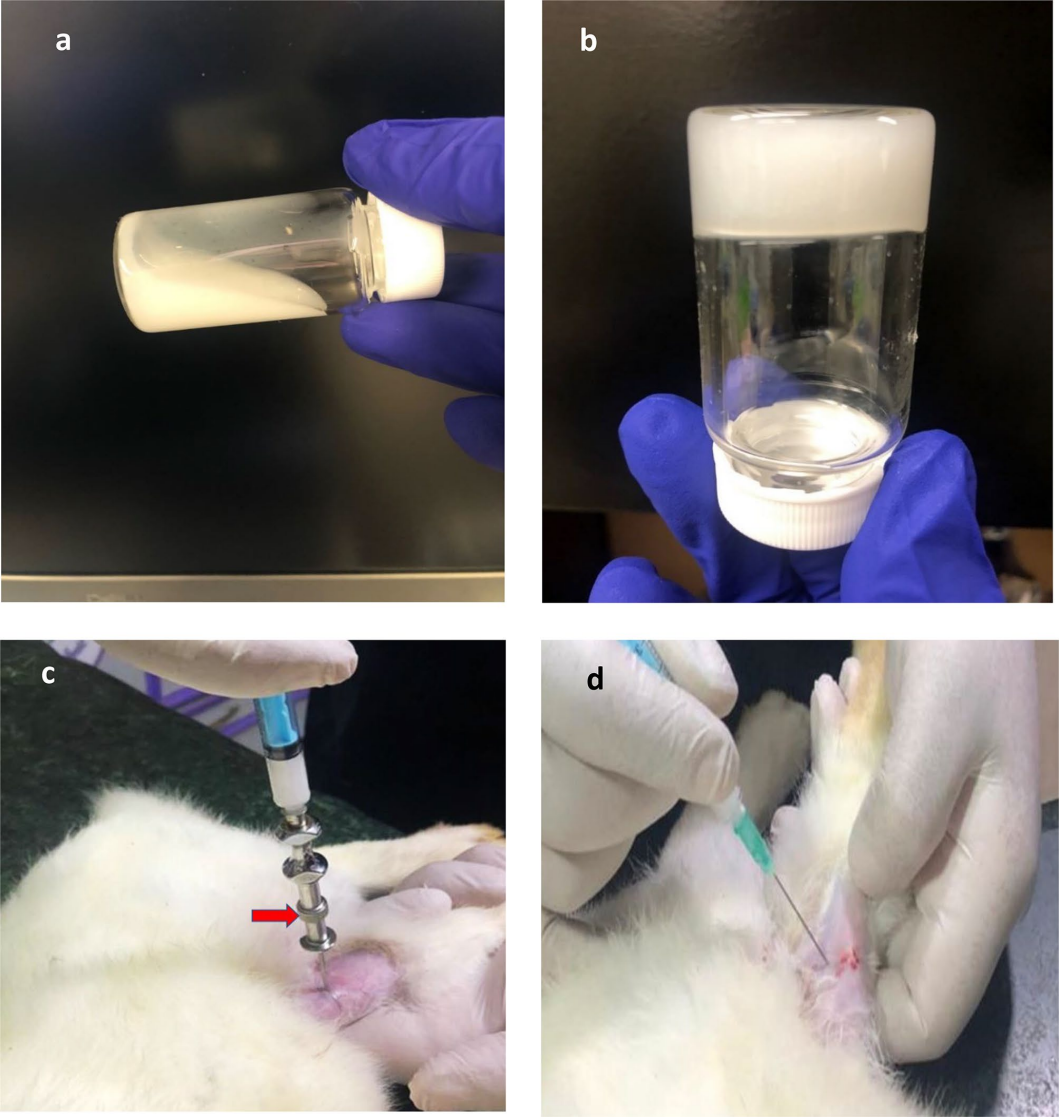
Photographic images showing: **a** flowable in situ forming hydrogel/nanoparticle-combined system ready for injection at room temperature, **b** gel formed after incubation at 37 °C for 2.5 min, **c** the injection of MRSA at the tibial metaphysis using bone marrow biopsy needle (arrow), and **d** the injection of the treatment system beneath the tibial shaft using 19-G hypodermic syringe

As presented in Table 3, it is clear that the incorporation of the spray-dried linezolid nanoparticles to the composite hydrogel significantly reduced the gelation time (*p* < 0.05) to half the recorded period for the composite hydrogel systems. This could be attributed to the fact that particulate system can assist the process of cross-linking between the polymers’ chains through physical interaction and/or adsorption on the surface of more than one polymer’s strings connecting them. Furthermore, particulate can limit the mobility of the polymer chain as a result of incorporation among their cross-linked chains and hence affecting their immobilization. These results are in agreement with those obtained in previous study [83] upon the incorporation of halloysite nanotubes into chitosan/glycerophosphate hydrogel. The obtained gelation times for the combined hydrogel/nanoparticle systems can be considered appropriate for clinical applications since the fast conversion into gel form guarantee holding the incorporated nanoparticles of the drug inside the in situ forming hydrogel matrix to be released over long period of time to the infected tissue. This would be advantageous provided that they are kept in refrigerator after mixing with NaHCO_3_ solution until the time of injection to guard against gelation before injection. Photographs of model hydrogel/nanoparticles combined system (H1NP3) at room temperature; 25 °C (flowable liquid) and at 37 °C (non-flowing gel) are illustrated in Fig. 3a and b, respectively.

#### In vitro drug release of linezolid from hydrogel/nanoparticle-combined systems

The results of the in vitro release testing of linezolid from the chitosan combined systems (H1NP3 and H1NP6) are presented in Fig. 1c. Fitting the release data to different kinetic models (zero order, first order, and Higuchi diffusion) and confirming the release mechanism through ap plying Korsmeyer-Peppas equation [45] revealed the diffusion model to be the best-fitting equation for the release of linezolid from which the time required for 80% release was calculated.

The two combined systems: H1NP3 and H1NP6 succeeded to sustain the drug release for 144 h and 240 h, respectively with significantly (*p* < 0.05) lower initial release after 30 min of 13.1% ± 0.47 and 12.57 ± 2.64, respectively compared to the release of the drug from both the composite hydrogel and the spray-dried nanoparticles. Moreover, the t_80%_ for H1NP3 and H1NP6 were 93.89 h and 166.28 h, respectively (Table 3), showing a significantly longer t_80%_ (*p* < 0.05) when compared to the release of the drug from any of the two systems separately. These results validated the combined in situ forming gel/ nanoparticle-combined system as a tool to clinically deliver the drug over much longer period of time when injected to the close vicinity of infected bones due to the additive sustainment effect on the release pattern of the polymeric nanoparticles and the formed hydrogel at 37 °C.

#### Measurement of pH of hydrogel/nanoparticle-combined systems

The pH of the two combined systems: H1NP3 and H1NP6, before the addition of NaHCO3 solution, were found to be 3.99 ± 0.02and 4.42 ± 0.01, respectively. This pH range guarantee the protection of the positively charged amino groups on the polymer chains of chitosan against neutralization, which keeps the electrostatic repulsion among the chitosan matrix and guard against precipitation. It was reported that chitosan is soluble and positively charged below its pKa value (6.2.) [84]. The pH of the combined systems upon the addition of NaHCO_3_ was recorded to be 6.76 ± 0.04 and 6.92 ± 0.02 for H1NP3 and H1NP6, respectively as presented tin Table 3, which assure transformed into precipitate-like gel when the temperature reaches 37 °C [85]. The previous findings prove the suitability of the combined systems for injection to the infected area as in situ forming gel provided that it is dispensed in the form of chitosan/nanoparticle-combined mixture and neutralizing NaHCO_3_ solution.

#### Scanning electron microscopy (SEM)

The SEM analysis of the lyophilized combined systems containing the drug in nanoparticles: H1NP3 and H1NP6 in comparison to the composite hydrogel, H1, containing the free drug was carried out and the photomicrographs are presented in Fig. 2c, d, and e, respectively. All three systems showed porous structures, which was more prominent in the hydrogel system containing the free drug (Fig. 2c). This may be due to the larger percentage of solid materials in the combined hydrogel/nanoparticle system than that present in the composite hydrogel which may deposit on the pores walls as shown in Fig. 2d, e, respectively. The porous structure of hydrogels was proven to be essential for the penetration of cells, replacing the aqueous dispersion medium, to permit cells proliferation and differentiation and for allowing the transport of nutrients to the repaired tissues [86, 87].

#### Effect of storage on the physicochemical characteristics of the combined systems

The optimized ready to be injected combined systems (H1NP3, H1NP6) stored at ambient temperature; 25 °C ± 3 °C in a preliminary study for a moth showed significant increase (*p* < 0.05) in viscosity (Table 3). Hence, a study for a period of 3 months was conducted on both systems in refrigerator temperature; 5 °C ± 3 °C both as a ready to be injected product and as two separate solutions (namely chitosan containing linezolid nanoparticles and nHA and NaHCO_3_ solution) where both forms maintained homogenous colloidal nature. They showed non-significant difference (*p* > 0.05) in drug content, the gelation time, viscosity, t_80%_, and pH (data not presented). Therefore, it is recommended that the combined hydrogel/nanoparticle system to be stored at refrigerator temperature either in a ready to be injected product or better as two separate solutions if longer storage period is needed before the time of injected.

#### Gamma sterilization

After gamma irradiation of the chitosan dispersion as well as NaHCO_3_ solution separately, they were mixed to obtain the in situ forming combined systems and were re-evaluated for their physicochemical characteristics. There was no significant difference (*p* > 0.05) in viscosity, drug content, in vitro gelation time, t_80%_, and pH after gamma irradiation (data are not presented).

#### In vivo animal study

##### Clinical evaluation

After injection of 0.1 mL of standardized MRSA dispersion in normal saline intramedullary to induce infection, as illustrated in Fig. 3c and the injection of the treatment systems at the area between the tibial shaft and posterior tibial muscle using a syringe with 19-G needle as presented in Fig. 3d, all experimental animals were clinically examined via body weight determination, rectal temperature measurements, appetite observation and signs of lameness during movement in the cage. No general health problems were observed in all animals; however, some animals showed septic rhinitis with nasal discharge that disappeared after the injection of treatment. There was also moderate limb lameness during the animal’s movement within the cage through the study period. The rabbits’ appetite was not changed during the experiment except for those in the control group (non-treated group), which showed slight decrease in their appetite. Animals lost 4 to 5% of their body weight during the induction of infection (4 weeks) and continued in losing weigh in the control group reaching 10–11% at the end of the experiment (8 weeks) while those in treated groups regained 50% of the lost weight after treatment. Regarding the rectal temperature, there was slight increase (0.2–0.8 °C) during the period of infection induction in all experimental animals. However, the temperature went back to normal level within 1–2 weeks post-treatment.

##### Radiographic evaluation

**Fig. 4:**
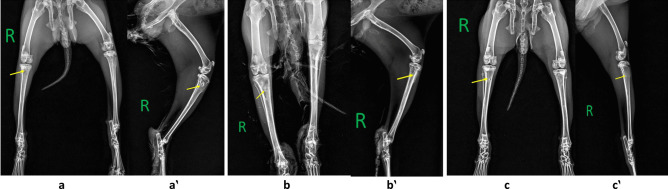
Radiographs showing: (**a**) pelvic limbs Ventrodorsally view and (**a‵**) right hind limb lateral view of an animal after 4 weeks of infection showing the injection site at the proximal tibia with radiographic changes (yellow arrow) manifested by moderate changes in medulla radiodensity and the cortex, (**b**) pelvic limbs Ventrodorsally view and (**b‵**) right hind limb lateral view of an animal from the control group (infected and not treated) after 8 weeks of infection showing the injection site at the proximal tibia with severe radiographic changes (yellow arrow) manifested by changes in medulla radiodensity and the cortex, (**c**) pelvic limbs Ventrodorsally view and (**c‵**) right hind limb lateral view of treated group with H1NP3 after 8 weeks of induction of infection (4 weeks after treatment) showing the injection site at the proximal tibia with no radiographic changes (yellow arrow) either in medulla or in cortex

Examination of the animal radiographically after 2 weeks post-infection induction showed no changes or signs of bone infection. It was not until the 4th week when signs of infections were detectable radiographically. At the 4th weeks post-infection, most of the animals showed moderate radiographic changes in the proximal tibia expressed as heterogenicity in medullary cavity radiodensity and little changes in cortical thickness and radiodensity (Fig. 4(a, a‵)). At 8th weeks post-infection in the control group (non-treated), the radiographs showed severe radiographic changes at the inoculation site as signs of sequestrum formation and cortical irregularities with soft tissue swelling (Fig. 4(b, b‵)). While in treated animals with all of the tested systems namely, H1, H1NP3 and H1NP6 (4 weeks post-treatment), there were no observable radiographic changes within the tibial bone nor soft tissue except the inoculation site was still obvious within the bone (Fig. 4(c, c‵)).

##### Laboratory assessment

###### Hematological features

For assessment of the inflammation condition during the induction of infection and treatment periods, white blood cell (WBC) count, erythrocyte sedimentation rate (ESR), and C-reactive protein (CRP) analysis were performed weekly after the injection of the MRSA suspension as well as the treatments under investigation.

**Fig. 5:**
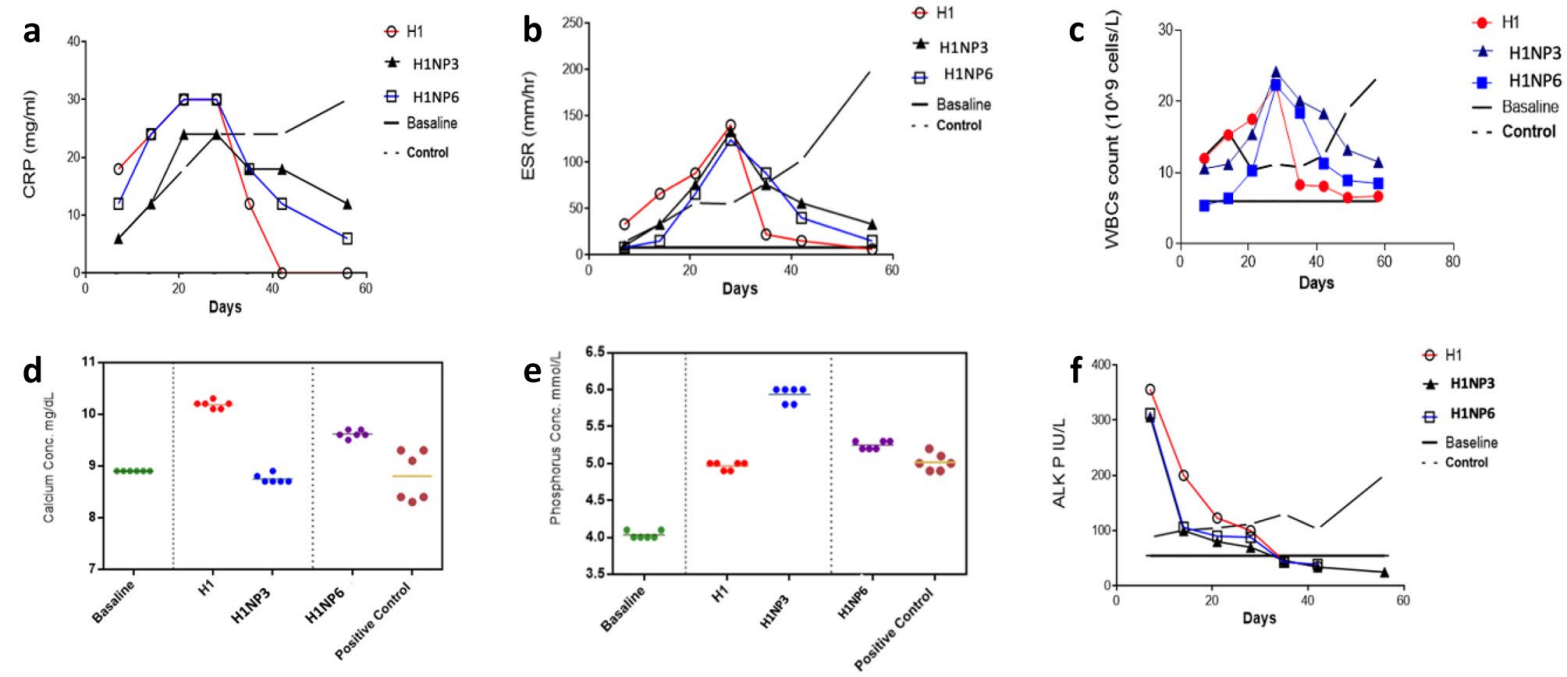
Hematological features; **a**, **b**, & c in the treated groups with composite hydrogel H1, hydrogel/nanoparticles combined system; H1NP3 and hydrogel/nanoparticles combined system; H1NP6 in comparison to base line and untreated control group: a C-reactive protein (CRP), b erythrocyte sedimentation rate (ESR), c white blood cell counts (WBC) and biochemistry parameters; d, e & f for treated groups in comparison to base line and untreated control group: d calcium, e phosphorus Sera distribution, f total alkaline phosphatase activity

All animals at the baseline before induction of infection had normal CRP and ESR readings, which were both noticeably lower than those associated with osteomyelitis. At day 7 after induction of infection, the 4 experimental groups of rabbits showed the development of increased CRP and ESR, which subsided by day 32 except the positive control which showed marked increase (Fig. 5a, b). These data are considered a point to an early acute phase of the illness. According to the literature, ESR and CRP levels are usually increased in acute infections [88]. There was significant difference (*p* < 0.05) in the rates of decline of CRP among the three experimentally treated groups. However, a non-significant difference in the ESR values was observed between H1NP3 and H1NP6, both were significantly different from H1. The results of the WBC count showed leukocytosis associated with acute stages of osteomyelitis. Normal rabbits had a WBC count of roughly 6.5 ± 10^^^9 cells/L at baseline. Due to the presence of bone infection, the WBC count was noticeably high (average 22.31.5 ± 10^^^9 cells/L) at the day before the injection of treatment in experimentally infected groups. After the injection of treatment (Fig. 5c), the WBC count steadily declined in the experimental groups, reaching a level that was similar to normal one after 3 weeks in group III receiving H1NP6, 2 weeks in group II receiving H1NP3, and 1 week in group I receiving H1, which is in direct correlation with the release rate of the drug from the three systems. This finding suggests that the composite gel can be used if fast treatment is required however, more frequent injections might be needed. On the other hand, the combined hydrogel/nanoparticle system may be of great value if longer therapy period is required where a composite hydrogel loading injection can be given to shorten the onset of action followed by as many as required injections of the combined systems to maintain the drug concentration at the site of injection.

###### Blood biochemistry

Serum levels of total calcium, phosphorous, and alkaline phosphatase were determined and compared with readings at baseline before infection (Fig. 5d, e, and f, respectively). The serum total calcium and phosphorus level was steady at all stages of experiment, where no significant elevation in comparison to serum total calcium and phosphorus in healthy animals was observed (Fig. 5e, f). However, there were variable changes in serum alkaline phosphatase (ALP) activity after induction of infection in the experimental groups in comparison to their baseline readings; there was no significant difference in its activity between the different experimentally treated groups in the current study. In metastatic and some metabolic bone disease, alkaline phosphatase, calcium, and phosphorous are elevated, but they are reported to be within normal limits in osteomyelitis [89].

##### Histopathological evaluation

**Fig. 6:**
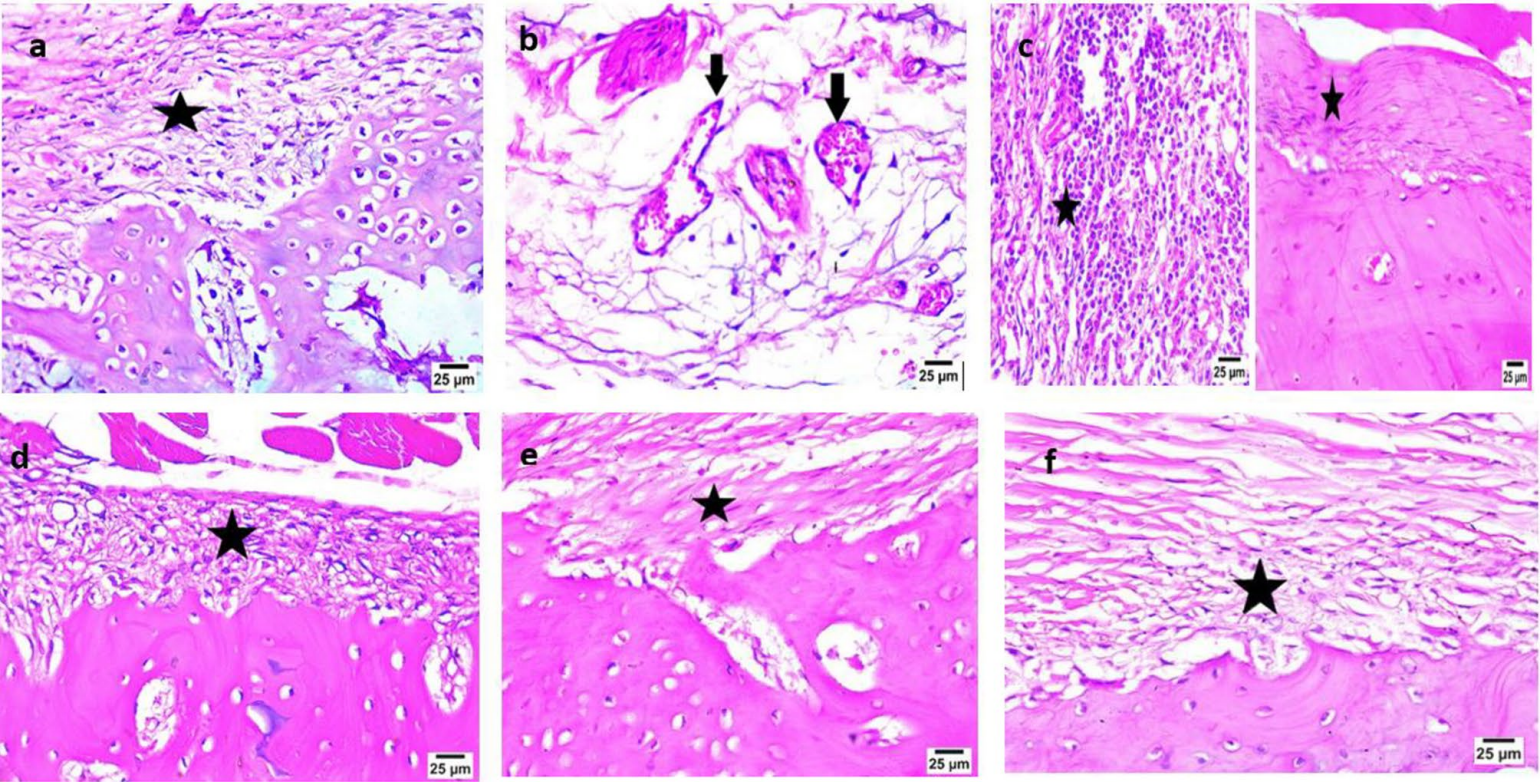
Photomicrographs (hematoxylin and eosin stain) showing **a** moderate fibrosis of periosteum (star) at 2nd week post-infection (arrow), **b** severe periosteal fibrosis with infiltration by mononuclear inflammatory cells with newly formed blood capillaries (arrow) in control animal group at 4 weeks post-infection, **c** severe periosteal fibrosis (star) with infiltration by mononuclear inflammatory cells with newly formed blood capillaries with degree of cortical bone thickening and necrosis in control animal group at 8th week post-infection, **d**, **e**, & **f** moderate periosteal fibrosis with infiltration by mononuclear inflammatory cells (star) in treated animal groups receiving H1, H1NP3, and H1NP6, respectively at 4 weeks post-treatment. The cortical bone and adjacent soft tissues and muscles showed no observable pathological changes

The development of acute osteomyelitis in the induced infected bones of this study was further confirmed by histological examination. Moderate fibrosis in periosteum without infiltration by inflammatory cells was observed at 2nd week post-infection (Fig. 6a). Sever fibrosis in periosteum with infiltration by high numbers of mononuclear inflammatory cells extended to the adjacent soft tissues and muscles together with the development of newly formed blood vessels were observed over the next 3 weeks of infections (Fig. 6b, c, & d). Furthermore, the cortical bone showed degree of necrosis and thickening in control group at the 4th and 8th weeks post-infection. While in treated groups, there were no specific differences in all treated groups of animals where they showed marked improvement in inflammation conditions and decreased inflammatory cells without cortical or medullary bone changes (Fig. 6e, f, & g).

## Conclusion

Compared to the systemic treatment, local administration of antibiotics could reduce the emergence of resistant* S. aureus* strains causing bone and joint infections as it has a direct effect on the site of infection and can avoid the side effects and drug toxicity if administered systemically. Based on our conducted studies, it was concluded that the optimized systems of the in situ forming chitosan composite hydrogel and the hydrogel/nanoparticle-combined system have pseudoplastics flow which favors their injectability and facilitate frequent administration to the close vicinity of infected bone, which can release linezolid over a week to 10 days which increase the patient compliance. They succeeded to alleviate the bone infections and the associated clinical, biochemical, radiological, and histopathological changes within 2–4 weeks of injection. We suggest that these systems can be very beneficiary as a solo treatment for patients in early stages of bone infections when no tissues debridement is required. They also can serve as supporting and stabilizing thereby in late stages of osteomyelitis after surgical debridement to maintain therapeutic level of linezolid at the surgical site and to assist the regeneration of the removed bone.

### Supplementary Information

Below is the link to the electronic supplementary material.

Supplementary file1 (DOCX 521 KB)

## Data Availability

All data, original sheets, and materials are available upon request.
