# Supplementary material for: Injectable systems of chitosan in situ forming composite gel incorporating linezolid-loaded biodegradable nanoparticles for long-term treatment of bone infections
Source: Drug Deliv Transl Res. 2023 Aug 4;14(1):80–102. doi: 10.1007/s13346-023-01384-x (PMC10746766; doi:10.1007/s13346-023-01384-x)
Supplement: Supplementary file 1 — Supplementary file1 (DOCX 521 KB) [file 13346_2023_1384_MOESM1_ESM.docx]

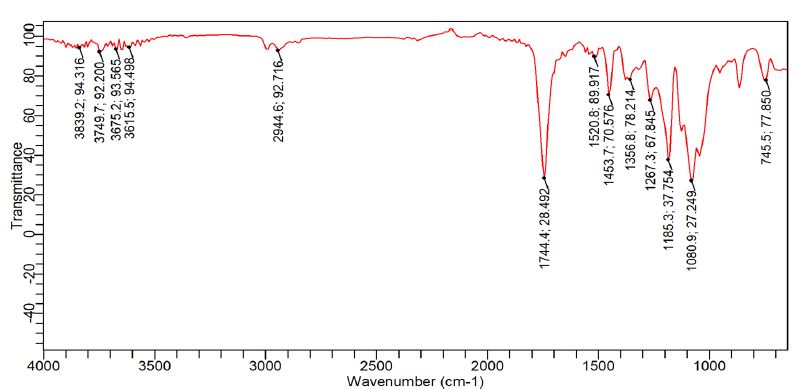

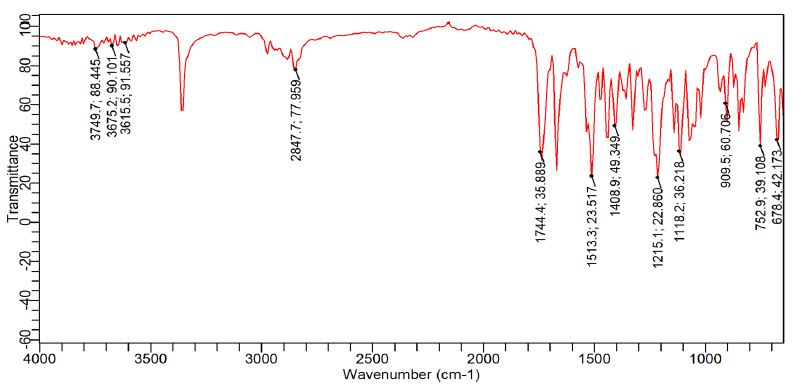


**a**

**b**

**c**

**d**

| 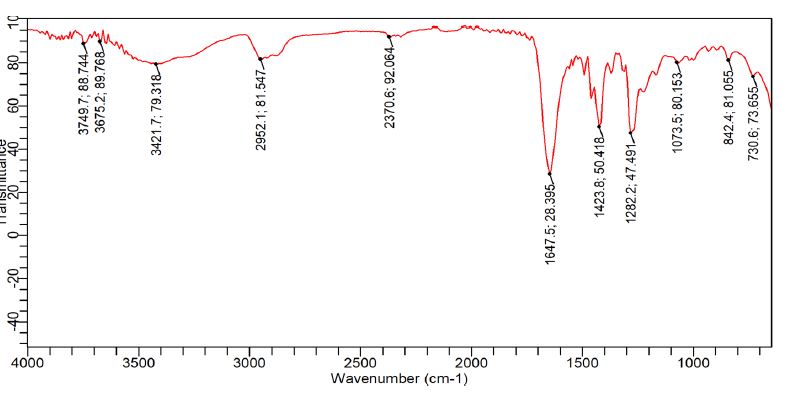 |
| --- |
|  |
| 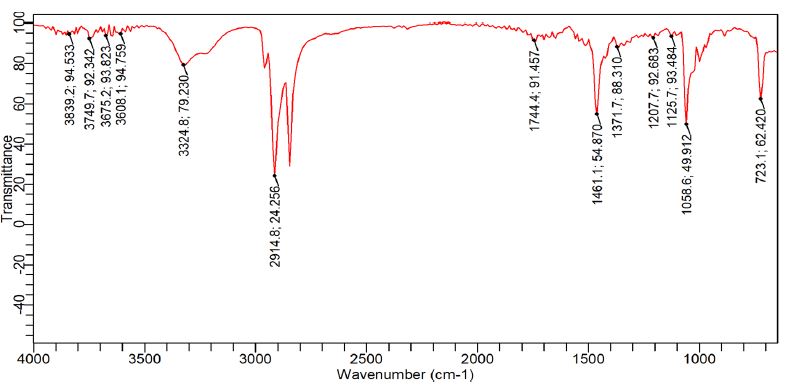 |
|  |
| 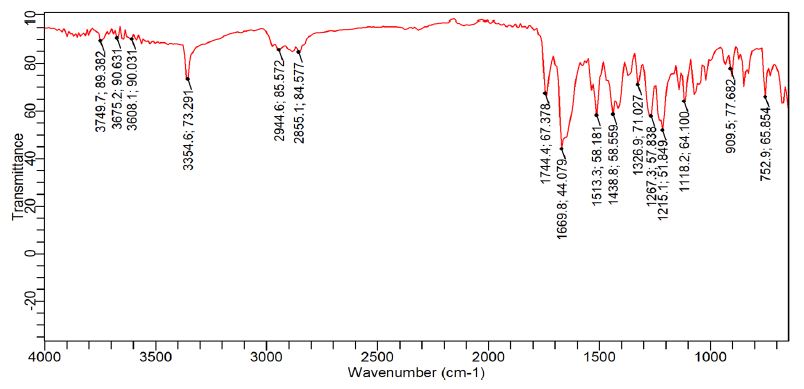  **e**  **f**  **g**  **h** |
| 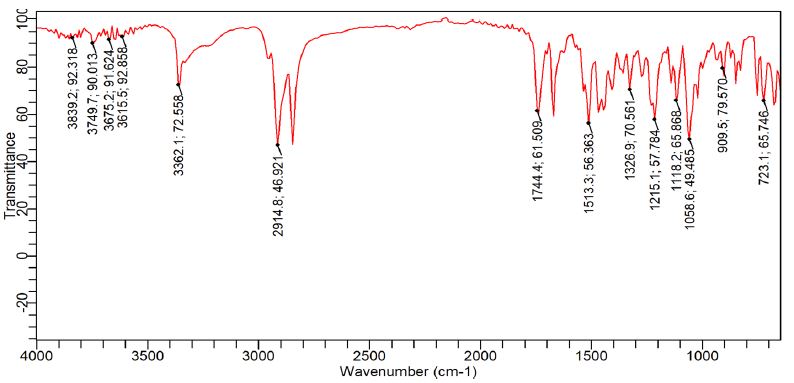 |
| 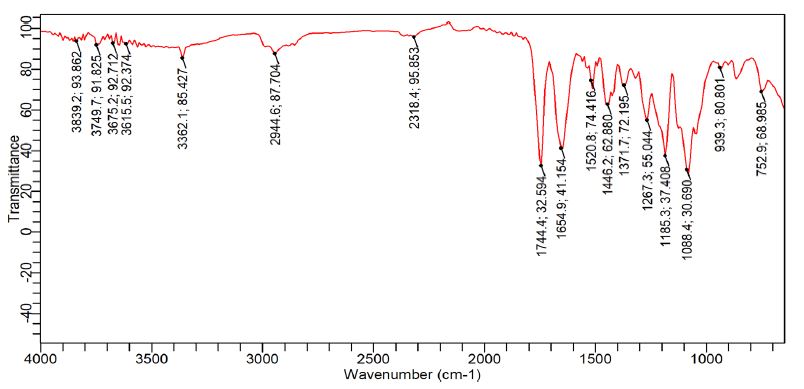 |
| 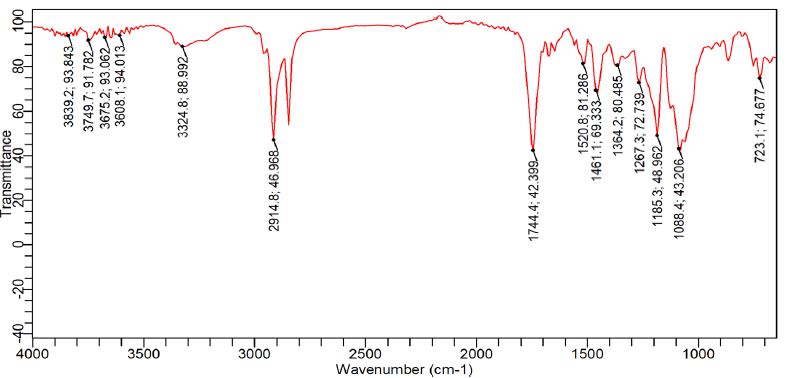 |

**Supplimintary-1. FT-IR spectra of: a) Linezolid, b) Poly-Lactied, c) PVP K30, d) Cetyl alcohol, e) physical mixture of NP3 components, f) physical mixture of NP6 components, g) NP3 and h) NP6.**


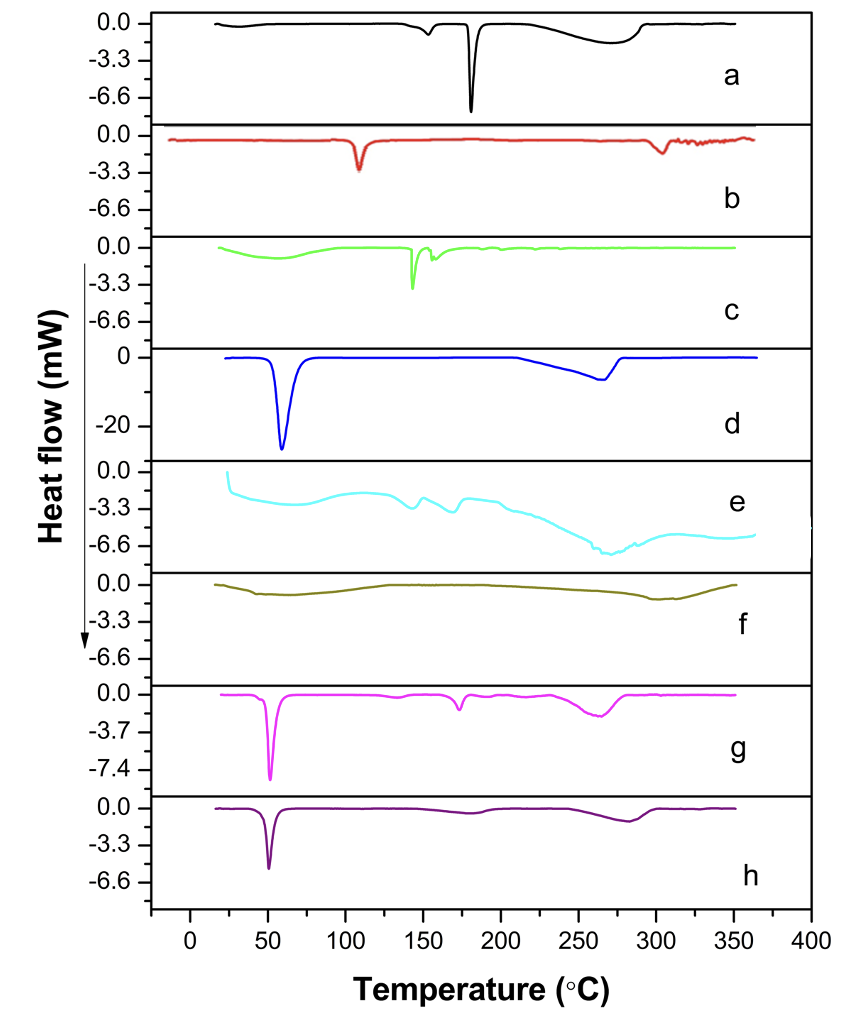


**Supplimintary-2. DSC thermograms of a) Linezolid, b) Poly-Lactied, c) PVP K30, d) Cetyl alcohol, e) physical mixture of NP3 components, f) NP3, g) physical mixture of NP6 components and h) NP6.**
